# 50-Years Inland Waterway Freight Data in the Rhine-Alpine Corridor

**DOI:** 10.1038/s41597-026-06875-3

**Published:** 2026-02-17

**Authors:** Bas Turpijn, Fedor Baart, Lóránt Tavasszy, Mark van Koningsveld

**Affiliations:** 1 Delft University of Technology, Faculty of Civil Engineering and Geosciences, P.O. Box 5048, 2600 GA Delft, The Netherlands; 2 Rijkswaterstaat, P.O. Box 2232, 3500 GE Utrecht, The Netherlands; 3 Van Oord Dredging and Marine Contractors B.V., P.O. Box 8574, 3009 AN Rotterdam, The Netherlands

**Keywords:** Geography, Environmental impact

## Abstract

To support a modal shift toward sustainable freight solutions, such as inland waterway transport (IWT), researchers and practitioners require long-term historical data on IWT freight flows. However, such comprehensive time series have been unavailable until now. This study addresses this gap by presenting a harmonized dataset encompassing 50 years (1970–2023) of IWT freight data across Europe, with a focus on the Rhine-Alpine Corridor. The dataset includes transport volumes (in tonnes) and transport performance (in ton-kilometers), classified according to NST-R, NST2007, and CCR nomenclatures. To ensure data continuity and completeness, processing techniques—including imputation and optical character recognition—were applied. The dataset offers valuable insights for researchers, policymakers, and transport planners aiming to comprehend and enhance the role of IWT in Europe’s freight transport landscape.

## Background & Summary

**Fig. 1:**
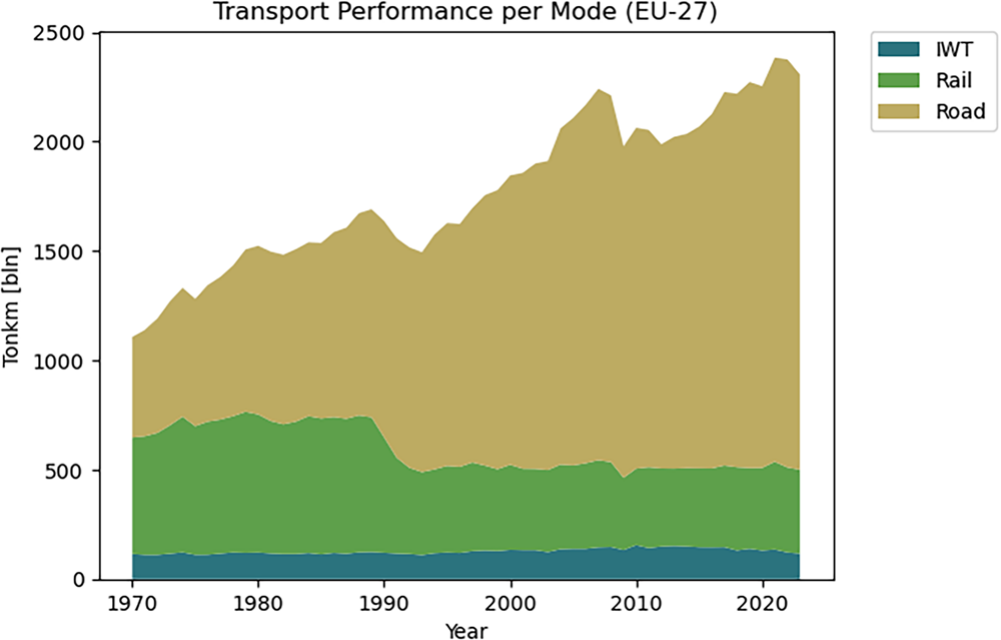
Overview of IFT performance in EU-27 per inland transport mode (Van Dorsser, 2025).

In recent decades, global freight transportation has grown significantly. In 1990, maritime vessels transported about 4 billion tons worldwide; by 2021, this figure had nearly tripled to 12 billion tons^1^. These developments had their impact on hinterland freight transport. Figure 1 illustrates the development of inland freight performance per transport mode within the European Union (EU) over the last 50 years^2^.

Since the fall of the Berlin Wall, in the early 1990s, inland freight performance in the European Union increased almost 50% in 30 years. Figure 1 makes very clear that most of this growth has been facilitated by road transport. As a consequence, many European countries are facing growing road congestion^3^ and environmental degradation because of increasing pollution, especially carbon dioxide^4^. Due to these developments, the European Commission has formulated ambitions to stimulate a modal shift to less polluting ways of freight transport^5^. Per ton-kilometer, waterway transport emits considerably less carbon dioxide (CO2) compared to other modes^6,7^. In addition, European inland waterways are not yet utilized to their full potential compared to roads and railways^8^. As a consequence, Inland Waterway Transport (IWT) is considered as a solution to accommodate future freight demand.

However, inland waterways also face challenges in realizing this modal shift ambition, partly due to its lack of resilience to disruptive events. Examples of such events in Europe were recently: ice formation in the Danube river^9^, extreme low water levels on the Rhine river in 2018 and 2022^10^, flooding^11^ and the COVID-19 pandemic^12^. These events had a significant impact on the reliability of the IWT system.

To explore the impact of modal shift policies, researchers and practitioners rely on detailed freight flow data to assess how the inland waterway transport (IWT) system responds to long-term trends and disruptive events. Gaining such insights requires access to consistent and comprehensive historical time series data. These time series are also essential for developing freight transport forecasts to plan for the future use of inland waterways. The quality of long-term forecasts for IWT can be severely hampered by lack of consistent time-series data and limited detail on cargo characteristics, as stated in two recent cases by^13^ and^14^. Spatial detail, as well as detail on cargo characteristics is crucial for understanding the dynamics of different IWT market segments.

Poor data availability and quality often hinder waterway authorities and researchers in generating reliable long-time series^15,16^. Especially data with detailed information on goods-types and origin–destination patterns are often not publicly available to researchers and practitioners^17^. If these data can be retrieved, often there are issues with completeness, consistency and accuracy^18^. Investments to overcome these data issues are therefore crucial to properly address the aforementioned data challenges.

This paper aims to fill these information gaps by providing goods-type information for the Rhine–Alpine Corridor and origin–destination information for inland waterway freight flows to, from, and via the Netherlands. We collected up to 50 years of IWT data in the Rhine–Alpine Corridor. This corridor accounts for about 70% of inland waterway transport in Europe^13^. The dataset comprises aggregated inland waterway transport (IWT) performance data (tonne-kilometres) for a large number of European countries, as well as IWT volumes (tonnes) by goods type for the Rhine–Alpine Corridor. In addition, for IWT volumes to, from, and via the Netherlands, origin–destination information for the past 25 years is included. The following text first addresses methodological choices, including data collection, classification, and processing. This is followed by a detailed description of the dataset and a summary of its contents. The manuscript concludes with notes on validation and potential uses of the data.

## Methods

This section outlines the methods used for data collection, classification, and processing. Data collection refers to how various input sources were acquired and integrated. Data classification focuses on the categorization of goods and the mapping between different nomenclatures. Data processing includes techniques to address data quality issues—such as missing values—and to harmonize heterogeneous data structures.

### Data collection

The IWT datasets within scope contain transport performance in ton-kilometres and transport volumes in tonnes for the main Rhine–Alpine Corridor countries: Belgium, the Netherlands, Germany, and France. In addition, we include IWT datasets for other European countries where available.

The transport performance dataset starts in 1970 and covers only the total aggregates per EU member state. It was derived from Van Dorsser^2^, who harmonized various inland freight transport datasets from EuroStat^19–21^, the OECD^22^ and several national statistical offices^23^.

**Fig. 2:**
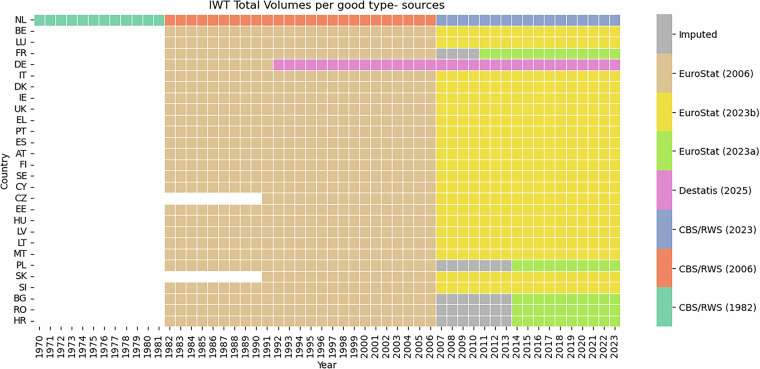
Overview of IWT data sources covering freight volumes per goods-type.

The transport volume dataset includes as much detail as possible on the types of goods. The Netherlands has the most detailed dataset on goods-type information. These data were provided by Statistics Netherlands (CBS) and the inland waterway authority (Rijkswaterstaat, RWS). For the period 1986–2023, this information is fully available in tabular datasets (CSV) and also includes the regions of loading and unloading^24,25^. Data from 1970–1986 had to be retrieved from historical archives (see Section Data processing). For Germany, the Federal Statistical Office (Destatis) has provided detailed goods-type information from 1992 onwards. Also this information is fully available in tabular datasets (CSV). For the other countries in the Rhine–Alpine Corridor, we collected IWT volumes by goods-type for the period 1982–2023 from Eurostat. Eurostat also provides these data for other EU member states, which we have therefore included as well. It is important to note that the IWT volume data are split in 2007, as a different goods-type classification was used in Europe prior to that year. The next section on data classification elaborates further on this matter. Figure 2 summarizes the availability and source of the IWT volumes per goods-type per EU member state.

Statistical offices, such as CBS, Destatis, EuroStat and OECD, arrange the access to these data, but they are not responsible for the data acquisition itself. Regarding the acquisition of IWT data, we distinguish two methods: collection along line infrastructure and collection at port infrastructure. In the first case, skippers have to report their trip, and cargo characteristics to the waterway authorities. Examples can be found in the Netherlands^26^, but also in the US^27^, where trip and cargo information are collected at locks and bridges and stored in traffic management systems. Another way to acquire cargo data from vessels is from the loading and unloading processes at ports. The German IWT statistics are derived from this process^28^. Both methods require further calculations to generate the datasets as provided by statistical offices. Trip data, collected at locks and bridges, need to be deduplicated to get unique trips for the entire network. Data from loading and unloading processes at ports on the other hand cannot provide direct information about transport flows on specific waterways. Then, traffic models are needed to assign the freight flows to a network^29^.

### Data classification

To add categorical information to freight data, classification schemes are needed. We harmonized the several classifications on goods-types and spatial units, which exist in the IWT data.

#### Goods-types

Several goods classifications exist worldwide. The US Army Corps of Engineers distinguishes 9 commodity groups in their statistical reports^27^, whereas the National Bureau of Statistics of China publishes IWT volumes for 12 distinct freight categories^30^. Since 2007, statistical bureaus in the European Union (EU) classify goods according to the Nomenclature Statistique des Transports (NST2007). This nomenclature distinguishes 20 goods groups, which can be subdivided into 81 sub-types. Another European classification scheme is the nomenclature of the Central Commission for the Navigation of the Rhine (CCR). The CCR distinguishes 8 goods-types in its annual market observations report, which are aligned with the main economic sectors^31^. The Dutch IWT management system acquires the goods-type information based on the international Harmonized System (HS) classification^32^. These HS codes are mapped at the sub-type level to the NST2007 nomenclature^26^.

Before 2007, a different nomenclature was used in the Europe: the NST-R. This nomenclature distinguished 52 goods groups and approximately 200 corresponding sub-types. Some member-states provide their IWT data at a highly detailed sub-type level, but most countries report only at the group level. As a consequence, it is complex to construct mappings between the several classification schemes and generate a consistent historical long-term time series per goods-type. Several attempts have been made to develop mappings between the nomenclatures (see^33–36^), but these lack completeness and/or present multiple key relations. The latter means that two or more goods-types in one nomenclature are related to two or more goods-types in the other nomenclature. A unique mapping is then not possible.

#### Goods-type correspondence tables

We combined the different correspondence tables from Eurostat^33^, Destatis^36^, Ports of Switzerland^34^ and Ports de Marchandises Françaises^35^. The Swiss correspondence table is the only one with unique mappings, but is not complete. We took this table as a base for our own mappings and added information from the German^36^ and French^35^ correspondence tables to produce a complete and unique mapping from the old NST-R nomenclature at sub-type level to the current NST2007 and CCR classifications. In cases where we still had multiple key relations, we chose the NST2007 sub-type with the largest share in that relation. And for the remaining NST-R sub-types without correspondence, we created a mapping to NST2007 sub-types based on expert opinion. The argumentation is also documented in the mapping.

The resulting mapping table can thus only be applied if goods are classified at sub-type level. Figure 2 presents the availability of IWT volumes per goods-type and per EU member state throughout time. Also the data sources are included. Table 1 summarizes the classification details per data source.

**Table 1:** Available good classifications and level of detail per data source.

Data Source	Good Classification	Spatial Units	Remarks
CBS/RWS (1982)	NST-R sub-types		Based on scanned documents. NST-R sub-types are mapped to NST2007 sub-types and CCR goods-types.
CBS/RWS (2006)	NST-R sub-types	Traffic Zones	Based on CSV files. NST-R sub-types are mapped to NST2007 sub-types and CCR goods-types. Traffic Zones are mapped to NUTS-2 regions
CBS/RWS (2023)	NST2007 sub-types	NUTS-2 regions	Based on CBS publicatiebestanden.
Destatis (2025)	NST2007 sub-types		Deliverd by Destatis.
EuroStat (2006)	NST-R groups		Downloaded from EuroStat: iww_go_atygo07.
EuroStat (2023a)	NST2007 sub-types		Downloaded from EuroStat: iww_go_atygo.
EuroStat (2023b)	NST2007 groups		Downloaded from EuroStat: iww_go_atygo.
Imputed	NST2007 sub-types		Imputed from EuroStat (2023a).

Only the Netherlands and Germany provide goods-type information at sufficient level of detail to construct a unique mapping between the several nomenclatures. For the other countries, the level of detail is not enough to map between the different nomenclatures. Therefore, we applied the mappings only to the Dutch dataset and generated a long time series per goods-type from 1970–2023 for the Netherlands. Destatis delivered the mapping to NST2007 sub-types, resulting into a time series from 1992–2023 for Germany. For France, Poland, Romania, Bulgaria and Croatia, we can present the IWT volumes per NST2007 sub-type level from 2007 onwards. The remaining countries provide IWT volumes only at the higher level of NST2007 goods groups.

#### Spatial units

Geographical information in Europe is typically structured according to the Nomenclature of Territorial Units for Statistics (NUTS). This hierarchical system consists of three levels:

- NUTS-1: major socio-economic regions
- NUTS-2: basic regions for the application of regional policies
- NUTS-3: small regions for specific diagnoses.

Origins-destination information in IWT data can only be published publicly on NUTS-2 level, due to privacy rules. In the Dutch datasets prior to 2007, however, origin–destination information per NST-R goods-type was recorded using so-called Traffic Zones. To construct a consistent long-term series—including NST2007 sub-types and origin–destination information at NUTS-2 level—we added a mapping from these Traffic Zones to NUTS-2. This enables a harmonised time series for the Netherlands from 1988 onwards.

### Data processing

Table 1 also provides an overview of the data-processing steps. Most datasets were either downloaded or supplied directly by the respective national statistical offices. The available tabular sources^23–25^ were straightforward to handle: the CSV files were imported into a database environment, from which the relevant goods-type classifications and IWT volume variables were extracted. Where available, origin–destination attributes were included in the selection as well.

Some data required additional processing steps. From 2007–2013 some discontinuities appeared in the IWT volumes on NST2007 sub-type level for France, Poland, Romania, Bulgaria and Croatia. These missing values are mainly a classification issue: the aggregates on NST2007 group level are all available. Using imputation techniques, we estimated the missing sub-type values and reconstructed complete time series for these countries. Since the group-level aggregates are known for all affected years, the overall data integrity is preserved. For the imputation, we applied an ARIMA-based approach to estimate the missing NST2007 sub-type values for 2007–2013. The imputed sub-type series were then scaled so that the sums exactly match the known group-level totals from the original data. This procedure yields consistent NST2007 sub-type time series for Poland, Romania, Bulgaria, and Croatia from 2007 onward—the introduction year of the NST2007 nomenclature.

The historical IWT data per goods-types in the Netherlands before 1982 are only available in scanned image documents. With help of optical character recognition techniques, such as the Python pytesseract library, we were able to process the scanned image documents. Although most goods-type information could be extracted automatically, missing or incorrectly recognised values were manually verified and corrected.

## Data Records

The datasets are available at Zenodo repository^37^. The IWT performance data set is named ‘eu_iwt_time_series_tonkm.csv’. The IWT volumes per goods-type is named ‘eu_iwt_time_series_goodtypes.csv’.

### Data definitions

We produced two IWT data sets: one file covers the total annual IWT performance (billion tonne-kilometers) per EU country from 1970 and the other includes annual IWT volumes (thousand tonnes) per EU country and goods-type. For the Netherlands, we included also the regions of loading and unloading on NUTS-2 level from 1988 onwards. The start year of the latter and the level of detail of the goods-types differ per country: Figure 2 shows the available data per country and year and Table 1 informs about the level of detail of the goods-type information, as well as spatial units. In the IWT volume dataset, we included the several available goods classifications. Unfortunately, the level of detail is not the same for every country. The Netherlands and Germany include goods-type information at sub-type level. With help of our generated mapping tables, we were able to convert the NST-R sub-types to NST2007 and CCR goods-types for these two countries (see Section Data classification). The other countries provide only goods-type information at aggregated group level. Table 2 summarizes the definition of the attributes in our dataset and the datatype.

**Table 2:** Data definitions per data set and attribute.

Data Set	Attribute	Definition	Data Type
IWT performance	land	Country where transport took place.	String
IWT performance	code	Country code.	String
IWT performance	corridor	Transport Corridor (Rhine, Danube, Other).	String
IWT performance	jaar	Year when transport took place.	Integer
IWT performance	prestatie	Amount of annual aggregated transport performance in billion tonne-kilometers.	Float
IWT performance	bron	Source of the data set.	String
IWT volumes per goods-type	jaar	Year when transport took place.	Integer
IWT volumes per goods-type	nuts2_laad	NUTS-2 region of loading.	String
IWT volumes per goods-type	nuts2_los	NUTS-2 region of unloading.	String
IWT volumes per goods-type	nstr	goods-type conform the NST/R nomenclature (only before 2007).	Integer
IWT volumes per goods-type	nst2007	goods-type conform the NST2007 nomenclature.	String
IWT volumes per goods-type	ccr	goods-type conform the CCR classification.	String
IWT volumes per goods-type	segment	Market segment (Dry Bulk, Liquid Bulk, Containers).	String
IWT volumes per goods-type	gewicht	Weight of the annual aggregated transport in 1000 tonnes.	Integer
IWT volumes per goods-type	land	Country where transport took place.	String
IWT volumes per goods-type	bron	Source of the data set.	String

### Data values

The data values are stored and published in CSV files and are available at Zenodo and Github (see Section Usage Notes). One record in the IWT performance data set represents the aggregated annual IWT performance of a country and one record in the IWT volume data set represents the aggregrated annual IWT volume per goods-type of a country. For the Netherlands, the regions of loading and unloading are also included from 1988 onwards. Transport performances can be summed, since they only cover the tonne-kilometers on national territory. Transport volumes cannot be summed over countries, due to duplicate data values between countries.

### Data overview

**Fig. 3:**
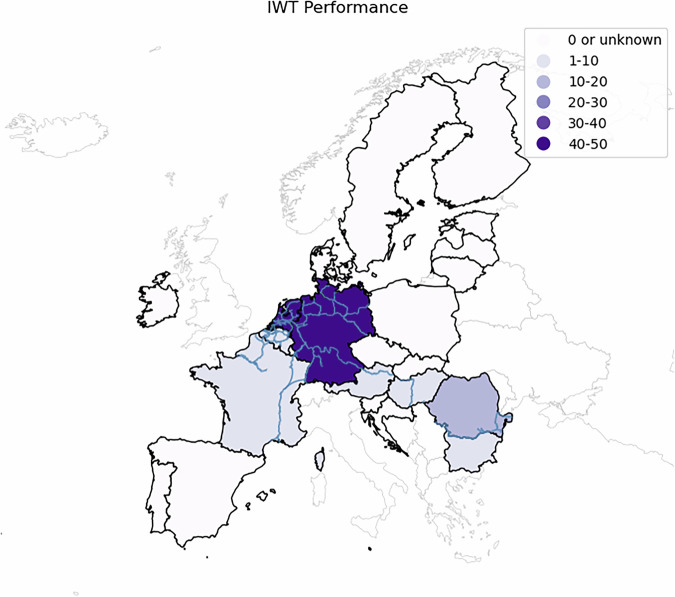
European IWT Performance (billion tonkm) in 2023 per European country (source: EuroStat, OECD).

The data product can be used to derive trends of IWT volumes and performance. The Dutch IWT volumes can also be used to perform spatial and network analyses. Figure 3 visualizes IWT performance in 2023 per country on a map. Around 70% of IWT activity in the EU takes place in the Rhine–Alpine Corridor, of which almost 90% occurs on the Dutch and German waterways. The inland waterway transport (IWT) performance in the Rhine-Alpine Corridor increased by approximately 7% over 50 years—from 98 billion tonne-kilometres (TKM) in 1970 to 105 billion TKM in 2020 (see also Fig. 1). In recent years, however, IWT performance in this corridor has declined. In contrast to this, the Danube–Black Sea Corridor shows an increasing trend.

**Fig. 4:**
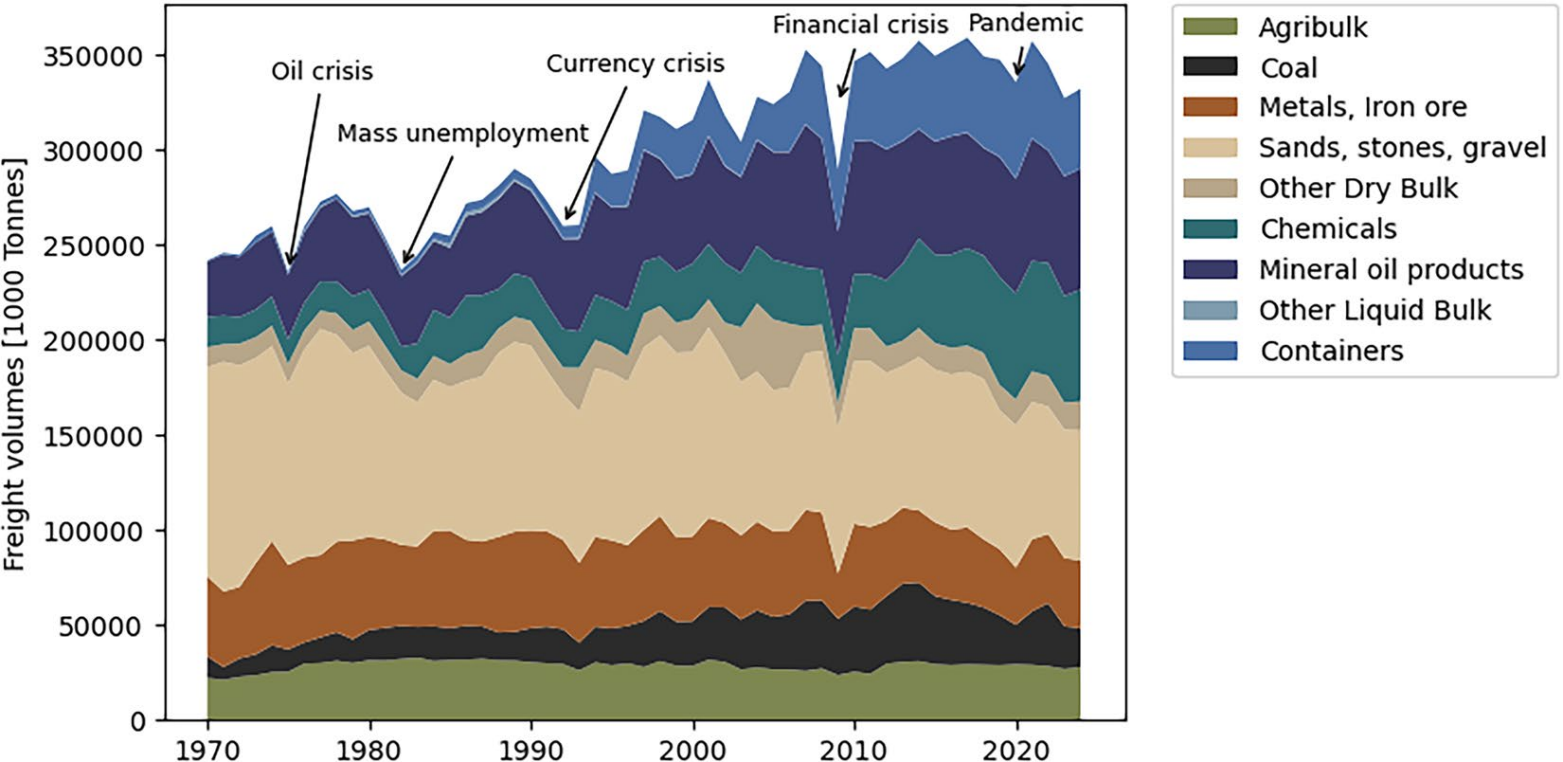
IWT Time series per CCR goods-type for the Netherlands (source: RWS, CBS).

To illustrate developments per goods-type, we present the 50-year IWT time series for the Netherlands. Figure 4 shows IWT volumes in tonnes by CCR goods-types. Over the past five decades, most of the growth has come from chemical products, mineral oil, and container transport. The latter evolved from a marginal segment in the 1970s into a key market today. The liquid bulk segment (chemical products and mineral oil) also expanded significantly: mineral oil volumes more than doubled; chemical products more than tripled. Agribulk grew by 25%. Coal volumes doubled compared to 1970 but declined by about 40% in the past decade due to German mine closures. Despite the recent decline, coal transport showed strong growth over a span of about 40 years. In contrast, construction materials (sand, gravel, stones) declined by over 30%, though still represent the largest category by weight.

## Technical Validation

We rely on the quality-assurance procedures applied by the national statistical offices and Eurostat, which include systematic checks for completeness, and potential missing values in the underlying datasets. In addition, we assessed the consistency of the Dutch and German data by performing cross-validation and network analysis.

### Missing values

For each year, the collected, classified and processed datasets (as described in Section Methods) were compared against the official annual IWT aggregates published by national and international data portals, including CBS (StatLine), Destatis (GENESIS) and Eurostat. This alignment step ensures that the constructed time series are consistent with national statistics.

For several Rhine–Alpine Corridor countries, notably France, total inland waterway transport volumes are not available before the early 1990s. In most cases, however, only the goods-type detail is missing: total annual volumes exist, but the underlying classifications required to derive goods-type breakdowns are absent. For these situations, we applied imputation procedures to reconstruct the missing goods-type values, as described in Fig. 2 and Section Data processing.

### Cross-validation

**Fig. 5:**
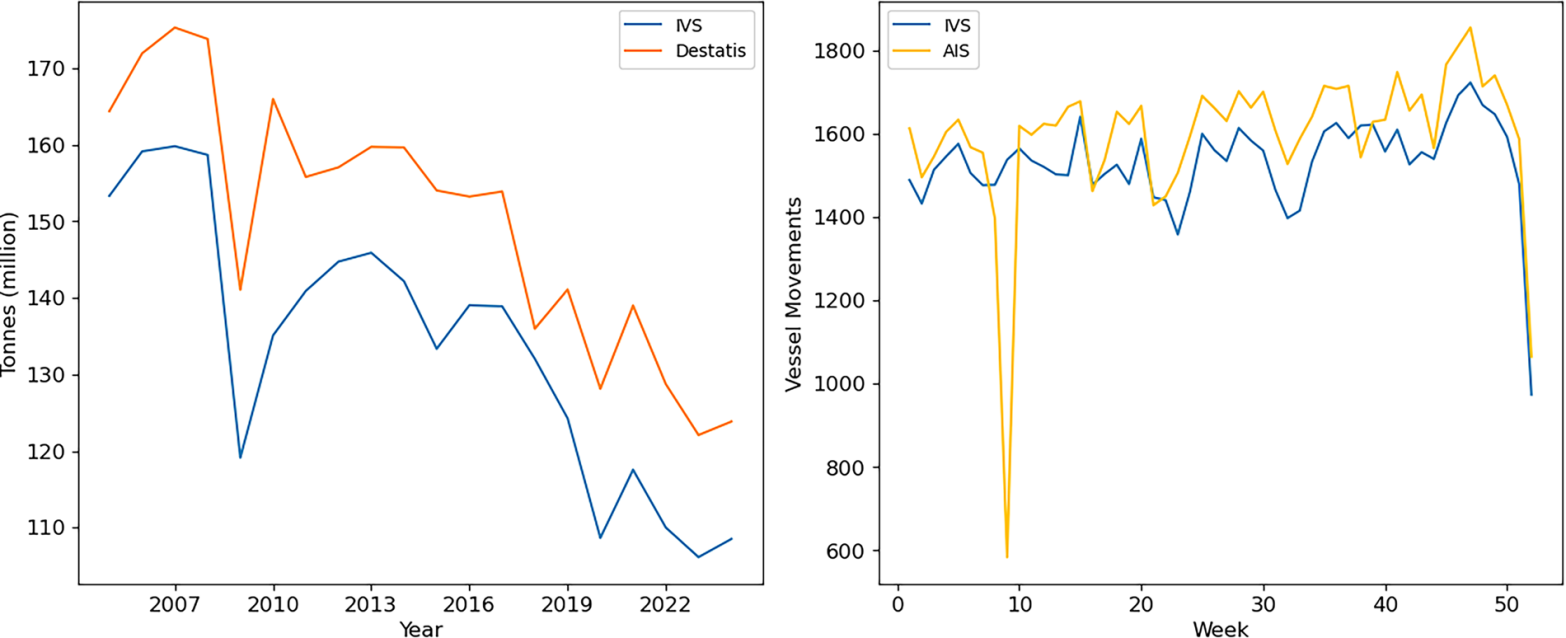
Rhine freight indicators at the Dutch- German border. Left figure: Annual transported volumes via Rhine at the Dutch-German border. Right figure: Weekly vessel movements via Rhine at the Dutch-German border in 2024.

In this study, cross-validation refers to the comparison of overlapping data from multiple independent sources to assess consistency. By example, the Dutch and German IWT data at the border should be the same. However, we observed some remarkable differences between the two sources. Figure 5 illustrates the annual IWT volumes via the Rhine river at the Dutch-German border between 2005–2024. The values from the Dutch Inland Waterway Transport Management system (IVS) are structurally about 10–20% lower compared to the German statistics by Destatis.

A comparison between vessel movements from the IVS and AIS tracks for 2024 confirms the observation that the Dutch IVS suffers from under registration of trip and cargo information (see Fig. 5). Although the AIS tracking data do not provide information about cargo volumes, these vessel movements can be used as an indicator for freight volumes^29^. The weekly patterns are quite similar, but the number of trips from the IVS are structurally lower than values from AIS.

Based on this analysis, our hypothesis is that the German data source is more comprehensive than the Dutch source, although additional research is required to confirm this. Despite the difference in completeness, both sources show comparable patterns. While the available data do not allow for a fully detailed assessment of inland waterway network usage in the Netherlands, they do support reliable trend analysis.

We supply the Dutch and German IWT volume datasets at the border from 2004 onwards, including goods-types at the NST2007 sub-type level. This enables practitioners to impute the missing cross-border IWT flows between the Netherlands and Germany according to their own analytical assumptions. Because the Dutch datasets also contain loading and unloading regions, practitioners can further refine and align specific cross-border flows between the two countries.

### Network analysis

**Fig. 6:**
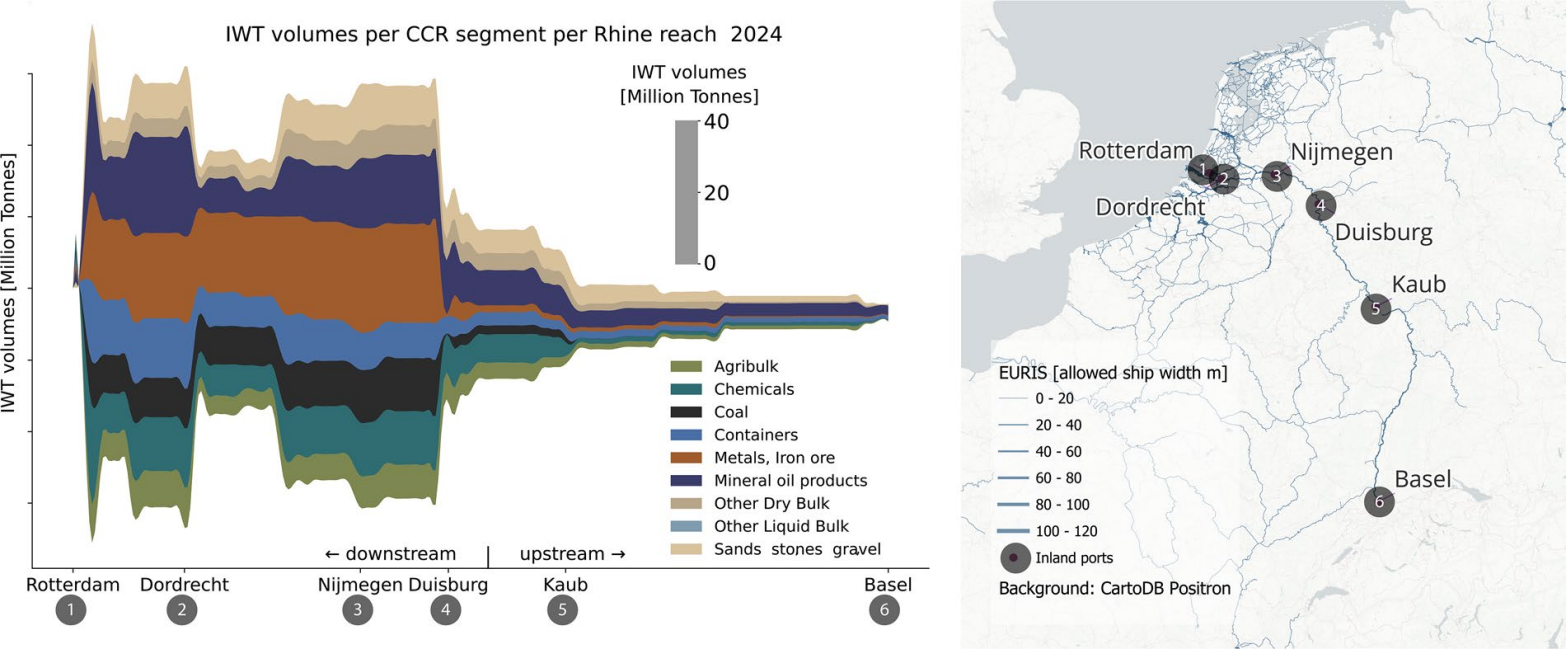
IWT volumes in 2024 per Rhine river reach and CCR goods-types.

We also examined whether the origins and destinations in our IWT time series reflect the spatial distribution of freight flows along the reaches of the Rhine. To this end, we assigned the Dutch part of the time series to the inland waterways based on the EURIS network. Origins and destinations were derived from NUTS-2 regions and mapped to their corresponding EURIS nodes. Using the Python library NetworkX, an optimal route (dimension restricted distance) along the inland waterway network was calculated for each origin–destination pair. After assigning the flows to the network, we aggregated the freight volumes by CCR goods-type and Rhine river reach. Figure 6 shows the results.

Key observations include:

1. **Rotterdam-Dordrecht**: These river reaches are closest to the port of Rotterdam. The Rotterdam–Duisburg flow represents the largest share, carrying metals, iron ores, containers, coal, and chemical products. Another important route runs between Rotterdam and Antwerp, where the transport of mineral oils plays a major role.
2. **Dordrecht-Nijmegen**: Downstream of Dordrecht, the Rotterdam–Antwerp flow branches off. Upstream of Dordrecht, the Antwerp–Germany flow converges with the Rotterdam–Duisburg flow, adding substantial volumes of mineral oil products.
3. **Nijmegen-Duisburg**: Downstream of Nijmegen, the Amsterdam–Germany flow merges with the other routes. Duisburg, Europe’s largest inland port, is the place where a very large share of inland waterway transport volumes is loaded or unloaded — particularly mineral oils, chemicals, and coal.
4. **Upstream of Duisburg**: These river reaches handle significantly lower IWT than the stretches downstream of Duisburg. Upstream of Duisburg, mineral oil and chemical products account for the largest share of the transported goods.

These key observations are consistent with regional economic information. The Ruhr area is the centre of the German steel industry, generating substantial transport flows of metals and iron ores. Since the closure of the coal mines in this region, coal has had to be imported from overseas, creating significant IWT flows between Rotterdam and Duisburg^38^. Chemical production is concentrated not only in the Ruhr area but also in upstream locations such as Ludwigshafen and the Neckar region^39^. Furthermore, total IWT volumes along the river reaches correspond well with the statistics reported in the CCR Market Observation^31^ and port publications^40,41^. Together, these findings support the spatial consistency of our IWT time series and the usability for network analysis.

### Limitations

As shown in Section Cross-validation, the reported IWT volumes crossing the Dutch–German border differ between the Dutch and German data sources. In theory, both sources should record identical values for this bilateral flow; however, differences may arise due to variations in data-collection practices. To support users in dealing with these uncertainties, we provide the border-crossing IWT volume series from both the Dutch and German sources. Access to both datasets enables researchers and practitioners to directly quantify the magnitude of discrepancies, evaluate their implications for empirical analyses, and select the most appropriate series for their specific application. Moreover, having two independent measurements of the same flow facilitates scenario development and sensitivity analysis. This dual-source approach enhances transparency and strengthens the analytical value of the dataset.

Between 2003 and 2006, a substantial share of iron ores in the Dutch dataset was misclassified as non-ferrous metal ores according to the former NST-R classification. As a result, the correspondence to NST2007 introduces inconsistencies at the sub-type level for this period. To ensure robust analysis, practitioners are advised to treat all ores as a single aggregated group (combining NST2007 sub-types 3.1 and 3.2) when working with the Dutch data for these years.

The origin–destination dataset is structurally limited to inland waterway freight flows that are loaded, unloaded, or transported via the Netherlands. Flows that neither originate, terminate, nor pass through the Netherlands are therefore not included in the origin–destination dataset, although they are recorded in the German dataset without detailed origin–destination information. This limitation should be taken into account when using the dataset for corridor-wide analyses beyond Dutch-related flows. However, according to the CCR Market Observation^31^, approximately 150 million tonnes of inland waterway freight were transported on the Traditional Rhine (German section) in 2024, of which about 105 million tonnes crossed the Dutch–German border (see also Fig. 5). Based on these figures, approximately 70% of Rhine–Alpine inland waterway transport volumes are directly related to the Netherlands and are thus included in the Dutch origin–destination matrix.

## Usage Notes

The datasets are archived on Zenodo and can be accessed via this DOI^37^. The IWT performance dataset is provided as ‘eu_iwt_time_series_tonkm.csv’, and the IWT volumes per goods type as ‘eu_iwt_time_series_goodtypes.csv’. The Zenodo page also includes a direct link to the corresponding GitHub repository, which contains additional details and the input data. The input datasets on GitHub include IWT volumes by NST-R goods types for each EU member state from 1982 to 2006 (‘estat_iww_go_atygo07.tsv’) and IWT volumes per NST2007 goods types between 2007–2023 (‘estat_iww_go_atygo.tsv’); these are also accessible via the Eurostat portal. The notebook ‘iwt_eu_cargotype_historical_imputated.ipynb’ is provided to generate the time series for most EU countries, including all imputations. The German IWT volumes per goods-type were delivered by Destatis, whereas the Dutch IWT volumes per goods-type were generated as described in Section Data collection.

We also included our generated mappings and the correspondences used in this study. The mappings from NST-R sub-types to NST2007 sub-types are provided in a JSON file. This file is named ‘mapping_nstr_nst2007_ra.json’ Each NST-R sub-type serves as a key, with values consisting of our own unique mapping to an NST2007 sub-type (NL) and the original correspondences from Germany (DE)^36^, France (FR)^35^, and Switzerland (CH)^34^. In addition, we included the mapping from NST2007 sub-types to the CCR goods-types, as well as the spatial mappings from the former Dutch traffic zones to the NUTS-2 regions. Both mappings are stored and published as JSON files, named ‘mapping_nst2007_ccr.json’ and ‘mapping_vgb_nuts.json’, respectively.

The data contains cargo information at an aggregated level, so there are no privacy issues or other limitations to freely utilize and adapt these data for all kind of research.

## Data Availability

The datasets are available at Zenodo repository^37^. Zenodo serves as the primary point of access. The output datasets are directly available there, while additional details, code, and processing scripts, including a flowchart (‘IWT_Data_Process.svg’) illustrating the folder structure and the location of input data (‘sources.zip’) and mapping files (‘mappings.zip’), are provided on the corresponding GitHub repository (see also Section Usage Notes). This structure allows readers to easily locate specific files, reproduce the analyses, and understand the organization of the dataset and supporting materials.

## Code availability

The code used to generate and analyze the data is written in Python 3.11 and runs in a Jupyter Notebook environment. The notebooks used to produce the datasets and the figures in this paper are available on Github, which can be accessed via Zenodo repository^37^. Specifically, see the file src.zip. For further details on the code and any additional manual processing steps, we refer to the README file.
